# The Biological Effects of Ivabradine in Cardiovascular Disease

**DOI:** 10.3390/molecules17054924

**Published:** 2012-04-30

**Authors:** Lorenza Speranza, Sara Franceschelli, Graziano Riccioni

**Affiliations:** 1 Department of Medicine and Science of Aging, University G. d’Annunzio, 66123 Chieti, Italy; 2 Cardiology Unit San Camillo De Lellis Hospital, Manfredonia, Foggia 71121, Italy

**Keywords:** heart rate, ivabradine, funny current

## Abstract

A large number of studies in healthy and asymptomatic subjects, as well as patients with already established cardiovascular disease (CAD) have demonstrated that heart rate (HR) is a very important and major independent cardiovascular risk factor for prognosis. Lowering heart rate reduces cardiac work, thereby diminishing myocardial oxygen demand. Several experimental studies in animals, including dogs and pigs, have clarified the beneficial effects of ivabradine associated with HR lowering. Ivabradine is a selective inhibitor of the hyperpolarisation activated cyclic-nucleotide-gated funny current (If) involved in pacemaker generation and responsiveness of the sino-atrial node (SAN), which result in HR reduction with no other apparent direct cardiovascular effects. Several studies show that ivabradine substantially and significantly reduces major risks associated with heart failure when added to guideline-based and evidence-based treatment. However the biological effect of ivabradine have yet to be studied. This effects can appear directly on myocardium or on a systemic level improving endothelial function and modulating immune cell migration. Indeed ivabradine is an ‘open-channel’ blocker of human hyperpolarization-activated cyclic nucleotide gated channels of type-4 (hHCN4), and a ‘closed-channel’ blocker of mouse HCN1 channels in a dose-dependent manner. At endothelial level ivabradine decreased monocyte chemotactin protein-1 mRNA expression and exerted a potent anti-oxidative effect through reduction of vascular NADPH oxidase activity. Finally, on an immune level, ivabradine inhibits the chemokine-induced migration of CD4-positive lymphocytes. In this review, we discuss the biological effects of ivabradine and highlight its effects on CAD.

## 1. Introduction

There has been increasing evidence that resting heart rate (HR) might be a marker of risk, or even a risk factor for cardiovascular morbidity and mortality. The relationship between resting HR and the development of cardiovascular disease (CAD), as well as all-cause and cardiovascular mortality, has been found to be strong and independent from other risk factors. Several laboratory studies indicate that HR plays an important role in the pathophysiology of atherosclerosis and in the clinical manifestations of coronary artery disease and that it is an independent prognostic factor in all cardiovascular syndromes [1,2,3]. Increased HR contributes to plaque rupture and to the downfall of myocardial ischemia distal to severe coronary stenosis with augmented cardiovascular mortality; vice versa, the HR reduction attenuates plaque rupture and decreases myocardial ischemia [4].

HR is a major determinant of myocardial oxygen consumption and of cardiac work. Thus, reduction of heart rate may represent an important strategy for the treatment of patients with a wide range of cardiac disorders.

Selective HR reduction by If-channel inhibition is a developed pharmacological principle in cardiovascular therapy [5,6]. The “funny” current, or “pacemaker” (I_f_) current, was described in cardiac pacemaker cells of the mammalian sino-atrial node (SAN). *I*_f_ was defined as the “funny” current because of several unusual features. One unusual feature is its voltage dependence. *I*_f_ is activated by hyperpolarization with a threshold of approximately −40/−50 mV in the SAN [7].

I_f_ is hence described as an inward current activated on hyperpolarization at voltages in the diastolic depolarization range, and contributing essentially to generation of cardiac rhythmic activity and its control by sympathetic innervation [8,9].

The channel activation is not modulated by a phosphorylation mechanism, but by cAMP binding. The cAMP, increase augments the plausibility of opening and thereby increase HR. Cholinergic stimuli differently decrease cAMP levels and reverse this process [10].

The molecular basis of If have been characterized by cloning a family of ionic channels known as HCN, which stands for Hyperpolarization activated Cyclic Nucleotide-gated channels [11,12]. 

Actually, four isoforms have been identified and cloned by different group, HCN1-4 [13,14]. This diversity could also be increased by the possibility that each isoform can co-assemble in heterotetramers, a process that has been suggested to be physiologically relevant [15,16].

HCN isoforms show characteristic properties of the native current If, as hyperpolarization activation, permeability to Na+ and K+, and modulation by cAMP and the block on the part of Cesium (Cs) [17].

Pharmacological agents able to interfere with the function of these channels have a potential therapeutic use in all those cases where it is useful to slow down and control the cardiac rhythm selectively without altering other cardiovascular functions such as ventricular contractility.

Several studies have shown that beta-blockers are able to reduce total mortality after myocardial infarction (MI). These beneficial effects have been imputed in part to the reduction of HR [18]. Beta-blockers not only reduce HR, but also have a number of other effects. They are used for the treatment of hypertension, congestive heart failure, atrial fibrillation, overactive thyroid, anxiety conditions, tremor, and of glaucoma [19].

Because of their multiple effects on different systems the beta blockers are not considered highly selective drugs for the therapeutic handling of heart disease associated with an alteration in HR.

Several molecules were developed to block the pacemaker channels, such as alinidine, cilobradine, zatebradina, ivabradine and ZD7288, but only ivabradine (a benzocyclobutane compound) has passed all the clinical trials and is currently marketed as an anti-anginal drug in different states, under the trade name of Procoralan [20,21,22,23]. Ivabradine represents the most specific and selective inhibitor for the pacemaker channels. Its beneficial effect is due to a selective slowing of the heart rate, without significant adverse actions such as reducing inothropism, a typical side effect of less specific inhibitors of rhythm, such as beta-blockers or calcium channel blockers. The inhibitory action of ivabradine on heart rate is due to a selective blockade of the “f” channels [24].

Ivabradine is the only drug currently available for the treatment of stable angina pectoris in patients that are intolerant of beta-blockers or in combination with beta-blockers to achieve heart rate goals [25,26,27]. The novel, specific HR-lowering agent ivabradine affects HR without directly altering other aspects of cardiac function [28]. Effectively, ivabradine acts on the SAN without the undesired effect of arterial vascular tone leading to hypotension [29].

By inhibiting the activity of the *I_f_* channel, ivabradine decelerates the gradient of diastolic depolarization reducing the pacemaker activity in the SAN. Because ivabradine does not influence contractility, it may also play a key role in the therapy of acute heart failure [30]. In addition, ivabradine has no contraindications of beta blockers in patients with respiratory failure and does not causes problems of asthenia [31,32]. Thus, ivabradine is the first, new class of agents that has HR-lowering properties, without other direct cardiovascular effects [33]. This molecule was also found to have beneficial effects on cardiac remodelling, capillary density and left ventricular dysfunction [34,35].

Already used for other cardiovascular disorders, ivabradine promises better oxygenation of the heart when it is subjected to a stress inducing a rapid, sustained and dose-dependent reduction of HR at rest and during exercise without a significant effect on atrio-ventricular conduction, left ventricular contraction/relaxation or vascular tissues [36]. These properties associated with an improvement in left ventricular loading related to bradycardia result in an increase in stroke volume and preservation in cardiac output even during exercise [37]. Various experimental and clinical studies showed the efficacy of ivabradine in patients with chronic stable angina, on heart rate reduction, on ventricular remodelling after acute myocardial infarction and on coronary blood flow [38]. 

Ivabradine is generally well tolerated [39]. The most common side-effect is phosphenes, symptoms related to the presence of a channel in the retina that is very similar to the HCN family. These visual symptoms are dose-dependent and resolve spontaneously during treatment or suspending treatment. Bradycardia has been reported in 2.2% of patients in clinical studies [40,41]. The safety of ivabradine has been documented in several studies and clinical trials. Unlike beta-blockers, ivabradine does not impair isovolumetric ventricular relaxation and does not exert a negative inotropic action. Therefore, the endothelium and left ventricular function are better preserved compared to beta-blockers [42].

## 2. Biological Effect of Ivabradine on Pacemaker Channels

Several biological studies have shown that ivabradine inhibits the pacemakers hyperpolarization-activated current in the SAN. The characterization of the blocking effect of ivabradine has also been extended to the HCN isoforms, HCN-4 and HCN-1 [43]. This revealed unexpected differences in the locking mechanism of the two isoforms.

In fact, electrophysiological studies conducted by Bucchi *et al*. in human embryonic kidney cells (HEK 293) co-transfected with human HCN4 (hHCN4) and mouse HCN1 (mHCN1) cDNA have established that the ivabradine is an ‘open-channel’ blocker of hHCN4, and a ‘closed-channel’ blocker of mHCN1 channels in a dose-dependent manner.

HCN4 block ivabradine-induced occurred only when HCN4 channel were open, indicating that the binding site is not accessible in the closed state. Contrariwise, ivabradine binds to HCN1 channels less favourably at depolarized than at hyperpolarized voltages when the cannel is in the closed configuration [44]. 

Studies of enzyme kinetics conducted by Thollon *et al*. showed that ivabradine inhibited the hHCN4 current in Chinese Hamster Ovary (CHO) cells in a manner dose-dependent with an IC_50_ of 0.54 µM. In order to analyse the possible implication of use-dependency in the kinetics of inhibition, the time-course effects of ivabradine have been compared with that of an immediate extracellular blocker of f-channels. Cs was used for this purpose as it blocks I_f_ channels in a non-use-dependent manner. The Cs (2 mM) induced the 90% of inhibition, at the first hyperpolarizing pulse, of the hHCN4 current and in 5–10 min exerted a very rapid reduction in HR of isolated SAN preparation. When about 60 pulses were required for an equivalent inhibition with 3 µM ivabradine and 90–180 min are needed to obtain a reduction of HR. These results suggest that the slow kinetics of action of ivabradine could be associated to its use dependent blocking mechanism and to the availability of open channels during the activating-deactivating pulses [45]. 

Genetic alterations of HCN4 channels are associated with cardiac rhythm disorders in humans. For example, a congenital mutation of a residue in the amino acid or protein HCN4 CNBD domain (S672R) is associated with congenital sinus bradycardia. 

*In vitro* studies indicate that the S672R mutation causes a shift in heterozygotes 5 mV towards more negative potential activation of the HCN4 channel, an effect similar to that of parasympathetic stimulation [46].

## 3. Biological Effect of Ivabradine on Endothelial Function

Endothelial dysfunction plays a major role in the cardiovascular disease, facilitating inflammation, platelet aggregation, and coronary vasoconstriction. Experimental data clearly suggest that endothelial dysfunction has pro-atherosclerotic and pro-thrombotic effects and has an important role in cardiovascular events. Variability in HR, associated with the development of endothelial dysfunction, may provide a new understanding of the basis for the association between increased heart rate and cardiovascular outcomes [47,48]. Treatment with ivabradine was demonstrated to improve endothelial function in several dyslipidemic mouse models with oxidative stress as a possible mediator for these effects [49,50]. 

Dyslipidemia induces endothelial dysfunction in which the damage from oxidative stress plays a significant role [51,52]. In dyslipidaemic mice, experimental data clearly show that the reduction of chronic heart rate with ivabradine preserves the function of the endothelium. Following treatment with ivabradine, a considerable improvement of vascular sensitivity to ACh is observed in the renal arteries, compared to WT mice with preservation of NO production. On the contrary, it has been observed that treatment with metoprolol the maximum dilator effect of Ach was retained, but is ineffective in preserving the cerebrovascular endothelial function. Furthermore, ivabradine normalizes the response of the endothelium to the antioxidant actions of N-acetylcysteine (at the level of the renal arteries), catalase and indomethacin (in the cerebral arteries). These experimental data show that ivabradine exerts its protective effect likely through an improvement of the shear stress-dependent stimulation of the endothelium, favouring eNOS expression and/or preventing NO or H_2_O_2_ degradation. There were no functional interactions with calcium channels and intracellular mechanisms that regulate the reactivity of smooth muscle cells, therefore the beneficial effects on endothelial function occur without alteration of the contractility of muscle cells [50,52]. Endothelial nitric oxide is a key mediator of endothelial function and atherogenesis [47]. Experiments conducted on aortic ring preparations from male C57/Bl6 mice (wild-type) and male ApoE^-/-^ mice evidence that ivabradine treatment improved the endothelial function through a reduction of vascular oxidative stress. Amelioration in endothelial function was correlated with a significant reduction in atherosclerotic plaque volume. RT-PCR and Western blotting analysis conducted on isolated aortic valve from ApoE^-/-^ mice in the organ bath treated with ivabradine show that the improvement in endothelial function by ivabradine was not associated with up-regulation of aortic eNOS mRNA expression, eNOS or p-eNOS protein level nor an increase of p-Akt expression. The observed effects are due to the eNOS-independent antioxidative effects of ivabradine. Furthermore, ivabradine had no effect on cultured endothelial and cultured vascular smooth muscle cells. In light of these experiments it is evident that a direct effect of ivabradine on vascular cells is unlikely, therefore, the primary mechanism of action of ivabradine is represented by the reduction of heart rate [53].

The pathogenesis of endothelial dysfunction and atherosclerosis is characterizes by release of reactive oxygen species (ROS) [54]. Treatment with ivabradine potently inhibited vascular oxidative stress, providing an explanation for the observed effects on vascular function. Superoxide derived from the vascular NADPH oxidase complex has been shown to impair endothelial function and to promote atherogenesis [55].

Histological studies by dihydroethidium staining has been revealed that reduction in HR ivabradine-induced was associated with marked inhibition of NADPH-oxidase activity and superoxide release. NADPH oxidase activity in the aorta was downregulated to 48 ± 6% in the ivabradine-treated ApoE^−/−^ mice. In addition, vascular lipid peroxidation as a global marker of oxidative stress was significantly decreased. The data of these experimental evaluations show a strong antioxidative effect of ivabradine on the vasculature. In addition, ivabradine treatment induces a potent downregulation of monocyte chemotactic protein-1 (MCP-1) expression.

MCP-1 contributes a link between endothelial dysfunction and atherosclerotic lesion formation by inducing leukocyte arrest and trans-endothelial migration. Moreover, MCP-1 has been shown to be regulated by fluid shear stress. 

Reverse-transcription PCR of aortic mRNA showed a marked reduction in MCP-1 expression to 26 ± 7% after treatment with ivabradine (10 mg/kg body weight per day for six weeks). On the contrary ivabradine had no effect on the aortic mRNA expression of vascular cell adhesion molecule-1 (VCAM-1) and intercellular adhesion molecule-1 (ICAM-1) [56].

In addition, selective HR reduction with ivabradine could be effective in the prevention and also in the treatment of erectile function ED in patients with cardiovascular diseases. ED is associated with the cardiovascular risk factors due to the crucial role of nitric oxide synthesis of the endothelial monolayer of the penile arteries and the corpus cavernous in the physiology of erection [57].

In hypercholesterolemic ApoE^−/−^-mice with consecutive impairment of endothelial, but also erectile, treatment with ivabradina induces a reduction of cavernosal oxidative stress and penile fibrosis. These benefits are associated with a reduced production of ROS in vascular NADPH oxidase activity reduced to two and the prevention of eNOS uncoupling. In contrast, in rats treated with angiotensin II, ivabradine slightly increased vascular oxidative stress, but did not affect endothelial dysfunction. Accordingly, the expression of components of the NO/cGMP pathway and NADPH oxidase subunits were not changed by ivabradine. The discrepancy between the observed ivabradine effects in the current study might relate to the different pathophysiological mechanisms leading to endothelial dysfunction in these models, the treatment duration or animal species.

During atherosclerotic disease, polymorphonuclear cells (PMN) invade the vasculature and their NADPH oxidase may also contribute to overall vascular oxidative stress. No effect on stimulated PMN was observed by different ivabradine doses, suggesting that this compound does not interfere with the ability of PMN to produce superoxide anions, and has no direct ROS scavenging properties either.

Long-term ivabradine treatment was able to increase the expression of components of the telomerase complex in the aorta, indicating decreased vascular cell senescence. Clearly, effects on vascular aging require longer treatment periods and therefore these findings may explain why vascular protection by ivabradine was only evident in ApoE knockout mice treated for 20 weeks [58].

Atherogenesis is an inflammatory process in the vessel wall involving inflammatory cells like monocytes, macrophages, and CD4-positive lymphocytes. A reduction in CD4 positive lymphocyte recruitment hampers lesion development and plaque formation [59].

A study performed by Walcher *et al*. demonstrates that, at an immune level, ivabradine inhibits chemokine-induced migration of CD4-positive lymphocytes. In CD4-positive lymphocytes stimulated with stromal cell-derived factor-1 (SDF-1), the pretreatment with ivabradine for 15 min reduces RANTES-induced migration, limits PI-3 kinase activity and phosphorylation of AKT, inhibits activation of Rac1 with subsequent inhibition of Myosin Light Chain (MLC) phosphorylation, reduces f-Actin formation, inhibits ICAM3 translocation and reduces SDF-1-induced trans-endothelial migration in a concentration-dependent manner [60]. Therefore, ivabradine inhibiting the migration of T lymphocytes in the vascular system, reduces the formation of pro-inflammatory cytokines with reduction of the development of atherosclerotic lesions. Further studies are needed to evaluate whether the effect of ivabradine on chemokine-induced migration *in vitro* could also be observed *in vivo*. 

## 4. Biological Effect of Ivabradine on RAAS

Heart failure (HF) is characterized by activation of renin-angiotensin-aldosterone system (RAAS). Elevated concentrations of angiotensin II associated with heart failure have been shown to promote atrial fibrosis through structural and electrical remodeling [61]. The improvement in cardiac function and fibrosis are dependent on the combined reduced levels of angiotensin and aldosterone, which act in synergy during cardiac remodelling [62].

To investigate the curative beneficial effect of ivabradine treatment on chronic and severe HF, Milliez *et al*. evaluated, in adult heart of male Wistar rats, the transcript level of the two main RAAS genes. Treatment with ivabradine cause a reduction in mRNA level and proteins level of cardiac ACE, marker of the activation of cardiac RAAS, and AT1 receptor. The decreased levels suggested that the low level of RAAS activation prevented the fibrotic remodelling of the remote myocardium. Thus, through its effects, ivabradine maintains cardiac function in HF [63]. In rat, AT2 infusion leads to hypertension, increased vascular oxidative stress due to an activation of the NADPH oxidase. Ivabradine does not reduce vascular ROS levels or NADPH oxidase activity in angiotensin II-infused rats and has no significant effect on AT2 signalling components in AT2-treated rats. Conversely, in a model of hypercholesterolemia-induced endothelial dysfunction angiotensin II serum levels and AT-1 receptor expression are decreased by ivabradine treatment [58]. This latter fact is also confirmed by other experiments conducted on DD-induced dyslipidemia in the hypercholesterolemic rabbit model, and it has been highlighted that circulating angiotensin II levels are significantly lowered by ivabradine and correlated with HR. Aldosterone levels also correlated with HR during treatment and were lower with IVA [64].

## 5. Conclusions

Cardiac rhythm disorders are associated with a high incidence rate and represent a major cause of mortality in Western countries. In recent years, molecular approaches applied to cardiovascular research have identified many genetic defects that lead to impaired cardiac function.

Several epidemiological studies have shown that elevated HR represents a risk factor for cardiovascular morbidity both in primary prevention and in patients with hypertension, CAD, and MI. Selective HR reduction by I(f)-channel inhibition is a pharmacological principle in cardiovascular therapy. Among the identified HR-lowering drugs, only ivabradine has now become approved for clinical use. 

Several studies show that ivabradine substantially and significantly reduced major risks associated with heart failure when added to guideline-based and evidence-based treatment. However, the biological effect of ivabradine have yet to be studied. Further investigations are therefore necessary to consider what are the mechanisms of action underlying the therapeutic effects of ivabradine, not only on the heart rate, but also on other systems directly or indirectly involved with the cardiovascular function.
